# Alterations in Inflammatory Markers and Cognitive Ability after Treatment of Pediatric Obstructive Sleep Apnea

**DOI:** 10.3390/medicina59020204

**Published:** 2023-01-19

**Authors:** Mohamed Shams Eldin, Mohamed Alahmer, Ebrahim Alkashlan, Mahmoud Zahran, Mohamed Eltonsy, Amr Zewail, Abdelfattah Kasem, Khaled Abdelaal, Mahrous Seddeek, Zakaria Ahmed

**Affiliations:** 1 Department of Otorhinolaryngology, Faculty of Medicine, Al-Azhar University, Cairo 11675, Egypt; 2 Department of Pediatrics, Faculty of Medicine, Al-Azhar University, Cairo 11675, Egypt; 3 Department of Clinical Pathology, Faculty of Medicine-Assiut, Al-Azhar University, Assiut 71542, Egypt; 4 Department of Anatomy and Embryology-Basic Sciences, Vision Medical College, Jeddah 7327, Saudi Arabia; 5 Department of Rheumatology and Rehabilitation, Faculty of Medicine, Al-Azhar University, Cairo 11675, Egypt; 6 EPCRS Excellence Center, Plant Pathology and Biotechnology Laboratory, Faculty of Agriculture, Kafrelsheikh University, Kafrelsheikh 33516, Egypt; 7 Department of Neurology, Faculty of Medicine, Al-Azhar University, Cairo 11675, Egypt

**Keywords:** obstructive sleep apnea, cognitive function, inflammatory cytokines, adenotonsillectomy, lifestyle intervention

## Abstract

*Background and Objectives*: Determination of the impact of obstructive sleep apnea (OSA) on the cognitive function (CF), and serum tumor necrosis factor-α (TNF-α), interleukin (IL)-6 and 1β levels and the effect of OSA management on these variables in children. *Materials and Methods*: A total of 224 patients were evaluated using the Pediatric Sleep Questionnaire, the NEPSY score for CF, and polysomnography (PSG) to grade OSA severity according to the apnea/hypopnea index (AHI). Adentonsillectomy (AT) was performed for patients with adenotonsillar hypertrophy grade > 2. Patients with overweight or obesity with mild or moderate OSAS underwent a 6-month protocol of lifestyle intervention (LSI). Blood samples were obtained for an enzyme-linked immunosorbent assay (ELISA) estimation of cytokine levels. All variables were re-evaluated at the end of the 6-month follow-up period. *Results:* A total of 181 patients had surgical interference and 43 patients underwent a LSI trial; 15 patients failed to respond and underwent surgery. At the end of the follow-up, 33 patients had residual OSAS with a significantly higher incidence among patients with severe OSAS, the mean score of the pediatric sleep questionnaire was significantly decreased in all patients, 181 patients showed an improved NESPY score, and cytokine levels were decreased. The baseline NESPY score, AHI index and sleep questionnaire score were negatively correlated. The percentage of change in the NESPY score and serum cytokine levels showed a positive correlation. *Conclusions:* OSAS is associated with cognitive dysfunction that significantly improves after adenotonsillectomy. LSI as a therapeutic line is satisfactory for children with mild OSAS and minimal cognitive dysfunction and is of value preoperatively to improve the surgical outcomes of AT.

## 1. Introduction

Sleep-disordered breathing is associated with sleep fragmentation and reduced blood oxygenation due to apnea and hypopnea episodes [1]. There is a significant incidence of obstructive sleep apnea (OSA) in children, which may have a variety of unfavorable effects on their health and behavior. Several studies have linked obstructive sleep apnea to serious medical problems in children, including epilepsy, hypertension, nocturnal enuresis, and failure to thrive. Multiple individual variables and systemic inflammation may influence the link between OSA and cognitive function (CF), which is currently being studied [2]. Multiple studies indicate that OSA can have a negative impact on CF, primarily the executive functions, attention, and episodic memory [1]. Childhood obesity is going to be a pandemic with a progressively increasing prevalence, reaching up to 5.6% in girls and 7.8% in boys as a worldwide prevalence, with much evidence supporting the assumption that obesity is a major preventable risk factor for some respiratory conditions, especially asthma and OSA [3]. The risk of pediatric OSA has been attributed mainly to the interaction of two considerations: BMI and tonsil/adenoid size. As a result, it was proposed that there are two forms of pediatric OSA, one associated with significant lymphoid hypertrophy in the absence of obesity (type I) and the other with obesity but only mild lymphoid hypertrophy (type II), with substantial overlap between these different entities. The global obesity epidemic has resulted in the development of a phenotypic variant of pediatric OSA in children that closely matches that of adults, along with a unique method for classifying pediatric OSA. [4] It was discovered that losing weight is an effective therapy for relieving OSAS; however, there was a large degree of variability in the improvement brought about by this treatment. Obesity exacerbates the effects of adenotonsillar hypertrophy (ADH), a common childhood condition that is a risk factor for obstructive sleep apnea. A change in BMI z-score is significant in pediatric OSA following AT, which suggests the valuable effect of this line of treatment [5]. The mechanisms that mediate the neurocognitive consequences of pediatric OSA includes intermittent hypoxia, arousal and sleep fragmentation besides the evolving role of a molecular basis responsible for end-organ damage including many recently studied biomarkers as plasma insulin growth factor-1 (IGF-1) plasma IL-6 and high-sensitivity C-reactive protein urinary neurotransmitters urinary catecholamines, taurine and GABA [6]. Interleukin-6 (IL-6), interleukin-1, tumor necrosis factor (TNF), and C-reactive protein (CRP) are examples of inflammatory biomarkers that have been found to be marginally related to a wide range of cognitive function indicators in overweight or obese children. These indicators include academic performance, executive function, behavioral functioning, and emotional functioning [7]. This study aimed to determine the impact of OSA on cognitive function and inflammatory mediators in children and to evaluate the effect of varied therapeutic modalities for OSA on these variables.

## 2. Materials and Methods

This prospective interventional clinical study was conducted in Ansari Specialized hospital and National Yanbu hospital KSA in the period from May 2020 to June 2022. All children aged 5–12 years who attended the outpatient clinics of otorhinolaryngology and/or pediatrics with complaints suggestive of OSAS were eligible for evaluation for exclusion and inclusion criteria according to the conditions of the Ethical Committee, which approved the study protocol. Children with mild to severe OSAS, free of exclusion criteria, were enrolled in the study after informed, written consent from their parents. Exclusion criteria included the presence of craniofacial anomalies, neurological disorders, very severe OSAS, OSAS complicated by complex comorbidities, residual OSAS following adenotonsillectomy, hypothyroidism, or refusal of the suggested therapeutic plans. Additionally, children who failed or were unable to undergo the cognitive function evaluation and those whose parents refused to undergo polysomnography were excluded from the study. The following evaluation tools were used for all participants in the study:

- Evaluation of body mass index (BMI): BMI was calculated as weight (kg) divided by the square of height (m^2^) and was interpreted according to the International Obesity Task Force (IOTF) BMI cut-offs according to the percentile of BMI adjusted for age and gender [8].
- Pediatric Sleep Questionnaire (PSQ) using the sleep-related breathing disorders scale, which consists of 3 domains including 22 items with three responses to each item: yes (=1), no (=0), and do not know = missed answer [9].
- Neurocognitive Assessments using the NEPSY II score which is designed to assess six domains, where each domain was expressed as a scaled score, with lower scores indicating cognitive dysfunction [10].
- Otorhinolaryngologic assessment variables:

a. Assessment of the volume of the palatine tonsils and adenoid size by the Brodsky grading scale [11] and X-ray soft tissue nasopharynx lateral view. Clinical grading of the examined upper airway was performed according to the modified Mallampati method [12].

b. Polysomnography (PSG) was performed according to the guidelines of the American Academy of Sleep Medicine (AASM) for the scoring of sleep and associated events in the Sleep Lab Unit of the Al-Ansari Specialized Hospital (Xltek^®^ Brain Monitor with Natus^®^ SleepWorks™ Middleton, USA PSG software) Overnight recording and scoring were carried out in accordance with the American Academy of Sleep Medicine’s 2020 guidelines [13]. Based on the apnea–hypopnea index values, patients were graded as mild if the AHI was 1–4.9, moderate if the AHI was 5–9.9, or severe if the AHI > 10; and if the AHI was >30, OSAS was very severe.

v. Laboratory investigation: venous blood samples (5 mL) were collected for ELISA estimation of serum levels of tumor necrosis factor-α (TNF-α), interleukin (IL)-6 and 1β.

Therapeutic plans were designed according to BMI, OSAS severity, and clinical evaluation. Patients with adenoid, tonsillar, or adenotonsillar hypertrophy of grade > 2 with clinically evident obstructive airways underwent adenoidectomy, tonsillectomy, or adenotonsillectomy. Overweight or obese patients with mild or moderate OSAS were randomly assigned to a 6-month trial of lifestyle intervention (LSI) and CPAP if indicated; responders spent more time in follow-up visits if surgical intervention was not indicated. For lifestyle intervention (LSI), a structured 6-month LSI program was costumed consisting of dietary modification and exercise. Intensive dietary counseling was provided weekly for the first 4 weeks of the intervention and monthly thereafter until 6 months. A target caloric deficit of 250.500 cal/d was recommended throughout dietary counseling. Dietary regimens consisted of diets composed of nutrients contributing to total energy as 55% carbohydrates, 15% protein, and 30% fat. Other lifestyle changes included calorie restriction based on snack consumption frequency, consumption of low-calorie and low-fat snacks, limitation of sugar-based carbonated drinks, and limitation of television viewing or mobile gaming duration. Exercise sessions consisted of both aerobic and strength training three times weekly. All patients were followed up for 6 months, either as part of watchful management and LSI or as a postoperative follow-up. At the end of the follow-up, all patients underwent evaluation for residual OSAS with PSG, a sleep questionnaire, CF using a NESPY score, and an estimation of serum levels of the studied cytokines. Obtained data were presented as the mean, standard deviation, numbers, percentages, median, and interquartile range. Results were analyzed using one-way ANOVA for the analysis of variance between groups, paired t-tests for the analysis of inter-group variance, and Mann–Whitney and Chi-square tests (X2 test) for the analysis of non-numeric data. Spearman’s correlation analysis was applied to evaluate correlations between baseline variables and between the percentages of changes in variable values at the end of the 6-month follow-up in relation to baseline values. The percentage of change was calculated as the difference between the baseline and end of the follow-up values, divided by the baseline value, and multiplied by 100. Statistical analysis was conducted using IBM^®^ SPSS^®^ Statistics (Version 25.0; IBM Corp., Armonk, NY, USA) for the Windows statistical package. A *p* value < 0.05 was considered statistically significant.

## 3. Results

**Figure 1:**
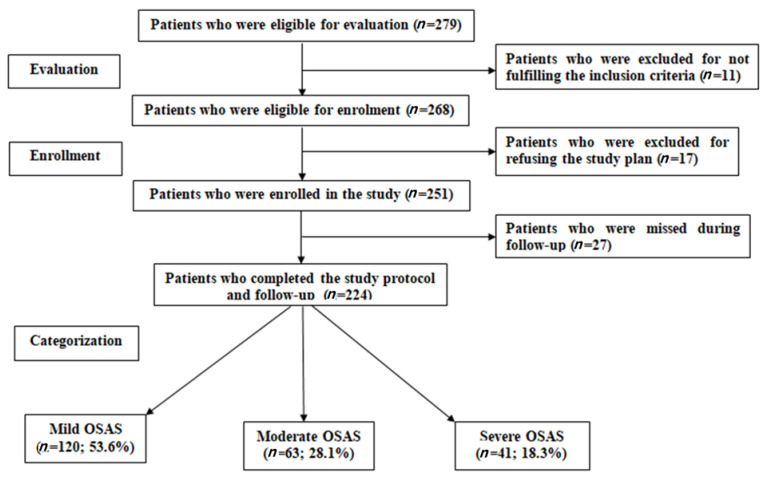
Study flow chart.

During the study duration, 279 patients were eligible for evaluation, 11 patients were excluded for not fulfilling the inclusion criteria, 17 patients refused to participate in the study, and 27 patients were missed during follow-up; thus, 224 patients were enrolled in the study and completed the study protocol and follow-up. As shown in Figure 1, patients were classified as mild (*n* = 120; 53.6%), moderate (*n* = 63; 28.1%), or severe (*n* = 41; 18.3%) based on their AHI score at the time of enrollment.

There was a non-significant difference between patients in the three groups with regard to age and gender distribution. However, the calculated BMI defined only 24 patients (10.7%) with an average BMI, while 75 patients (33.5%) were overweight, and 125 patients (55.8%) were obese. The BMI of patients with mild OSAS was significantly lower than the BMI of patients with moderate (*p*1 = 0.0001) and severe (*p*1 = 0.0001) OSAS, with patients with moderate OSAS having a non-significantly (*p*2 = 0.902) lower BMI than those with severe OSAS. Otorhinolaryngological examination defined 62 patients (27.7%) with grade 4 tonsillar hypertrophy, 99 patients (44.2%) with grade 3, 61 patients (27.2%) with grade 2, and only two patients (0.9%) with grade 1 tonsillar hypertrophy. The difference between the three groups was non-significant. As regards adenoid volume grading, 12 patients (5.4%) had no adenoid hypertrophy, 13 patients (5.7%) had adenoid hypertrophy of grade 1, 92 patients (41.1%) had grade 2, 81 patients (36.2%) had adenoid hypertrophy of grade 3, and 26 patients (11.5%) had adenoid hypertrophy of grade 4, with a non-significant difference between the three groups. Modified Mallampati score class IV was detected in 9 patients (4%), class III in 31 patients (13.8%), class II in 71 patients (31.7%), and class I in 113 patients (50.4%), with a lower frequency of patients having high Mallampati grades among patients with mild OSAS, and the difference was non-significant (*p*1 = 0.074), but was significantly (*p*1 = 0.0175) higher in comparison to the frequencies detected in patients with moderate and severe OSAS. High Mallampati grades showed a non-significantly lower frequency among patients with moderate OSAS than those having severe OSAS (Table 1).

**Figure 2:**
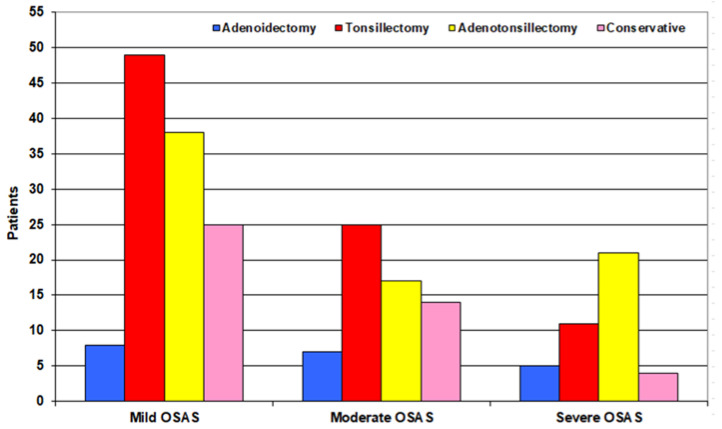
Patient distribution according to the surgical decision.

One hundred and eighty-one patients with adenotonsillar hypertrophy of grades 3 or 4 underwent an adenoidectomy (*n* = 19), tonsillectomy (*n* = 76), or adenotonsillectomy (*n* = 86), without trial of LSI or CPAP. Forty-three patients underwent the LSI trial for 6 months: 25 patients with mild, 14 patients with moderate, and 4 patients with severe OSAS, and 15 of these patients’ received CPAP in addition to the LSI protocol. Unfortunately, 15 patients failed to respond; 7 had mild, 5 had moderate, and 3 had severe OSAS and underwent surgery, while the remaining 28 patients succeeded in responding. With conservative treatment, the surgery was avoided, yielding a success rate of 65.1%. A total of 196 patients were surgically treated. The frequency of patients requiring a tonsillectomy or adenotonsillectomy was significantly higher among patients with severe OSAS but was non-significantly higher among patients with moderate OSAS in comparison to patients with mild OSAS, with a non-significantly lower frequency among patients with moderate OSAS, than among those with severe OSAS (Figure 2).

At the end of the follow-up, 33 patients had residual OSAS, with a significantly higher incidence of residual OSAS in patients with severe OSAS in comparison to its incidence among patients with mild OSAS (*p*2 = 0.001). Additionally, there was a significant difference between patients with mild and moderate OSA (*p*1 = 0.04), with a non-significantly (*p*3 = 0.092) higher incidence of residual OSAS among patients with severe OSAS than in patients with moderate OSAS. The pediatric sleep questionnaire baseline score was significantly higher in patients with severe OSAS compared to patients with mild (*p*2 = 0.002) and moderate OSA (*p*3 = 0.005), with a non-significantly (*p*1 = 0.692) higher score in patients with moderate OSAS compared to patients with mild OSAS. At the end of the follow-up, the mean score was significantly (*p*4 < 0.001) decreased in all patients in comparison to their baseline score. However, the score determined at the end of the follow-up was still significantly higher in patients who had severe OSAS in comparison to those who had mild (*p*1 = 0.002) and moderate (*p*2 = 0.047) OSAS, with a non-significantly (*p*1 = 0.192) higher score in patients with moderate OSAS in comparison to patients with mild OSAS (Table 2). Regarding cognitive function, the baseline NESPY score was significantly higher in patients with mild OSAS in comparison to patients with moderate (*p*1 = 0.004) and severe OSAS (*p*1 = 0.001), with a non-significantly (*p*2 = 0.096) higher NESPY score in patients with severe OSAS in comparison to patients with moderate OSAS. At the end of the follow-up, all patients showed a significantly (*p*3 < 0.001) higher NESPY score in comparison to their baseline score. At the end of the follow-up, the NESPY score of patients with severe OSAS was significantly (*p*1 = 0.030) higher in patients with mild OSAS in comparison to patients with severe OSAS but was non-significantly (*p*1 = 0.137) higher than the NESPY score of patients with moderate OSAS, while patients with moderate OSAS showed a non-significant (*p*2 = 0.798) higher NESPY score in comparison to patients with severe OSAS. The NESPY score at the end of the follow-up was significantly higher in all patients in comparison to their baseline score (*p*4 < 0.001). Differentially, at the end of the follow-up, 43 patients (19.2%) showed no change in NESPY score, while 181 patients (80.8%) showed an improved NESPY score, with a non-significantly higher frequency of patients showing an improved NESPY score among patients with severe OSAS in comparison to patients with mild or moderate OSAS. However, the percentage of improvement of the NESPY score was significantly higher in patients with severe OSAS in comparison to those with mild (*p*2 < 0.001) or moderate OSAS (*p*3 = 0.002), with a significantly (*p*1 = 0.002) higher percentage of improvement in patients with moderate OSAS compared to patients with mild OSAS (Table 2).

Baseline serum levels of inflammatory cytokines were significantly higher in patients with severe OSAS in comparison to patients with mild and moderate OSAS, with significantly lower levels in the serum of patients with mild OSAS than those with moderate OSAS. All patients, irrespective of OSAS severity, showed significantly lower serum cytokine levels at the end of the follow-up in comparison to their baseline levels. At the end of the follow-up, serum levels of TNF-α showed a non-significant difference between studied patients, irrespective of their baseline severity of disease. However, the percentage of decreased serum levels of TNF-α was significantly higher in patients with severe OSAS in comparison to patients with mild (*p*1 < 0.001) and moderate (*p*2 = 0.002) OSAS, with non-significant differences between patients with mild or moderate OSAS. As regards the percentage of change in serum IL-6 levels estimated at the end of the follow-up, it was significantly lower in patients with mild OSAS in comparison to that detected in levels estimated in the sera of patients with moderate or severe OSAS (*p*1 = 0.005 and 0.002, respectively), but was a non-significantly lower percentage of change in patients with moderate OSAS than in patients with severe OSAS. On the contrary, the percentage of change in serum levels of IL-1β in patients with moderate OSAS was non-significantly lower than that in patients with mild OSAS (*p*1 = 0.146) but was non-significantly higher than that in patients with moderate (*p*2 = 0.207) OSAS, with a significantly (*p*1 = 0.002) lower percentage of change in serum IL-1β in patients with severe OSAS than in patients with mild OSAS (Table 3).

TNF-α and IL-1β serum levels correlated positively and significantly with baseline BMI, whereas IL-6 serum levels correlated insignificantly. Additionally, the baseline AHI index showed a positive and significant correlation with baseline BMI and serum levels of the studied cytokines. Similarly, the baseline sleep questionnaire score showed a non-significant correlation with baseline BMI and serum levels of TNF-α and IL-6, but not with serum levels of IL-1β. The baseline NESPY score had a negative significant correlation with baseline BMI and serum levels of IL-6, but the correlation was non-significant with serum levels of TNF-α and IL-1β (Table 4).

**Figure 3:**
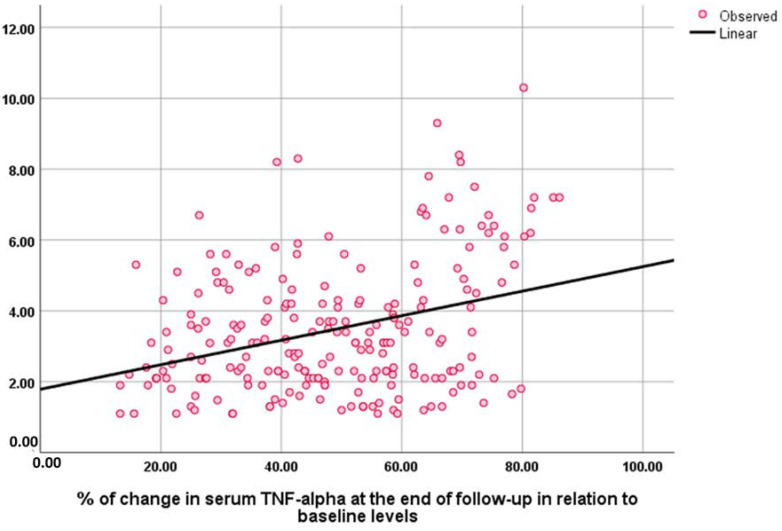
Correlation between the percentage of change in NESPY and serum levels of TNF-α at the end of the follow-up in relation to baseline levels.

**Figure 4:**
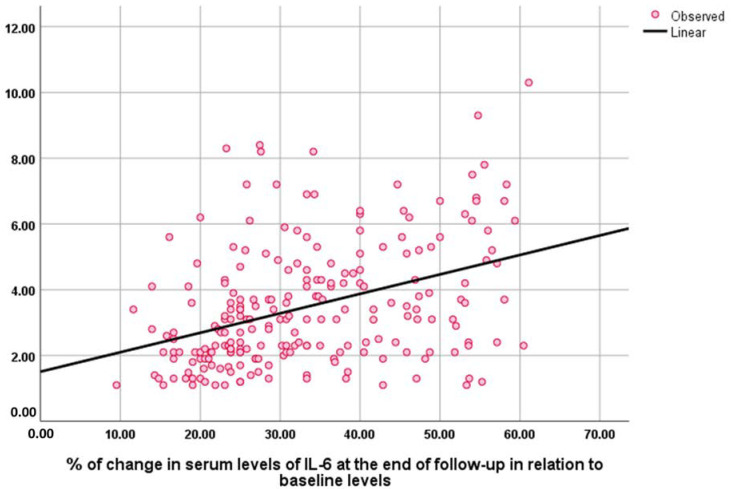
Correlation between the percentage of change in NESPY and serum levels of IL-6 at the end of the follow-up in relation to baseline levels.

**Figure 5:**
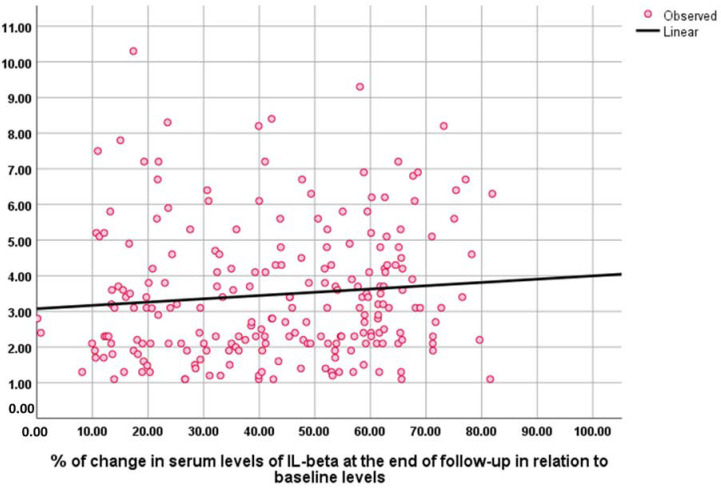
Correlation between the percentage of change in NESPY and serum levels of IL-1β at the end of the follow-up in relation to baseline levels.

The baseline NESPY score was negatively correlated with the baseline AHI index (Rho = −0.428, *p* = 0.001) and sleep questionnaire score (Rho = −0.196, *p* = 0.003). The percentage of change in NESPY score at the end of the study correlated positively and significantly with the percentage of change in serum levels of TNF (Rho = −0.248, *p* = 0.001), IL-6 (Rho = −0.196, *p* = 0.001), and IL-1 (Rho = −0.149, *p* = 0.026) (Figure 3, Figure 4 and Figure 5).

## 4. Discussion

The current study detected an evident impact of OSAS on cognitive function (CF), as evidenced by the improvement of CF assessment after management of the underlying cause of OSAS, and such improvement was found to inversely correlate with the severity of OSAS. Similarly, multiple studies detected such an effect of OSAS on neurocognitive functions, where mild to moderate childhood OSAS was found to adversely affect CF, particularly in children younger than 6 years of age [14]. Furthermore, other studies documented that OSAS is often complicated by cognitive dysfunction manifested in executive function, attention, memory and learning, especially in children [15]. On the other hand, it was found that school-aged children included in a wide community-based research study with a higher AHI showed significantly lower cognitive performance but with subtle statistically significant differences in office-based cognitive tests, and attention was specifically affected in habitual snorers with normal polysomnographic indices [16].

However, the effect of AT on the neurocognitive functions was a matter of discrepancy, where no differences found between AT and watchful management for OSAS on CF in children and AT may have limited benefits in reversing any cognitive effects of OSAS [17]. Thereafter, surgical treatment in school-age children did not lead to improvements in objective attention measures parallel to improvements in polysomnography (PSG) and parent-reported symptoms [18]. On the contrary, the results of the current study concerning the improved CF after AT support a meta-analysis reporting that OSAS children perform worse than healthy children in all cognitive domains, but after 6–12 months following AT, significant improvement in attention–executive function and verbal ability were reported in comparison to their baseline level, with restoration of attention–executive function and memory in comparison to healthy children [19]. In another review of the literature, 11 studies investigated changes in behavior and cognitive outcomes after AT, and almost all of them reported a significant improvement of the scores after AT [20].

Recently, improved anxiety and decreased auditory/visual sustained attention abnormalities in children with OSAS after AT were documented [21]. The cause–effect relationship between adenotonsillar hypertrophy and OSAS and the impact of AT on OSAS manifestation are evident in the current study by the significantly lower scores in the pediatric sleep questionnaire (PSQ) after management of the underlying pathology for OSAS in comparison to the baseline score. Thereafter, improvements in sleep and behavior following AT have been detected by PSG monitoring and parental questionnaires [22].

Interestingly, the current study detected significantly higher baseline serum levels of inflammatory markers in OSAS patients in comparison to age- and BMI-matched non-OSAS children and reported a significant decrease in these levels after AT with or without LSI in comparison to baseline levels. Similarly, significantly higher levels of inflammatory markers were documented in patients with OSAS than in OSAS-free people, with significant associations with increased BMI [23]. Serum levels of IL-6, IL-8, IL-17, IL-18, CRP, and TNF-α were also reported to be significantly higher in patients with OSAS [24].

Moreover, higher CRP was prospectively associated with increased OSA risk, and these abnormally high CRP levels detected in OSAS children were significantly reduced after AT [25]. The obtained data and review of the literature point to a possible causal relation between high serum inflammatory markers and development and severity of OSAS. In line with these data and suggestions, the levels of TNF-α and IL-6 and their gene polymorphisms have been found to be significantly related to the susceptibility to OSAS [26]. Furthermore, the current study detected a positive and significant correlation between baseline serum levels of inflammatory cytokines and AIH as a measure for OSAS severity. In support of these findings, recently, high serum levels of IL-6 and CRP were detected in OSAS patients, and these elevated levels were found to be parallel to the OSAS severity, confirming that OSA and inflammation are interconnected [27]. Additionally, it was found that elevated CRP levels are connected with a higher risk of neurocognitive impairments in pediatric OSAS. In addition, the inflammatory response, as demonstrated by TNF-α, may indicate the existence or absence of OSAS-related excessive daytime sleepiness. Changes in the urine concentrations of a variety of neurotransmitters have been also found to be associated with cognitive impairments in OSAS in children [28]. Another form of support for this interrelation is the persistent and significantly higher serum inflammatory markers in patients with residual OSAS (AHI > 1 event/h) after AT, and this could be attributed to still-present obesity, despite the trial of LSI. The relation between improved OSA and obesity treatment has been documented, and a significant reduction in AHI after 6 months of BMI z-score reduction has been detected [29]. Improved cognitive functions after AT with or without LSI could be attributed to the reported decreased serum levels of inflammatory cytokines, as evidenced by the inverse relation between the percentage of improvement in NSPY score and the percentage of decrease in serum levels of inflammatory cytokines, and this defines a vicious circle consisting of OSA with subsequent sleep disorders, elevation of serum inflammatory cytokine levels and cognitive dysfunction.

## 5. Conclusions

OSAS is associated with cognitive dysfunction that may be secondary to altered levels of inflammatory cytokines or as an impact of apnea/hypoxia episodes during sleep. AT significantly improved OSAS manifestations, cognitive function, and reduced serum cytokine levels. LSI is a satisfactory therapeutic line for children with mild OSAS and minimal cognitive dysfunction, and it is required preoperatively to improve AT outcomes.

## Figures and Tables

**Table 1:** Demographic and clinical data studied patients who were categorized at the time of enrolment according to the estimated AIH score.

Group Variables (mean ± SD)		Mild OSAS (*n* = 120; 53.6%)	Moderate OSAS (*n* = 63; 28.1%)	Severe OSAS (*n* = 41; 18.3%)	Significance of Difference between Groups		
					*p*1	*p*2	*p*3
AHI		3.2 [2.7–3.7]	7.9 [6.9–8.6]	14 [12.7–16.7]	-	-	-
Age (years)		8.3 ± 2	8.7 ± 2	8.3 ± 2.1	0.859	0.983	0.546
Gender	Males	64 (53.3%)	39 (61.9%)	24 (58.5%)	0.267	0.563	0.731
	Females	56 (46.7%)	24 (38.1%)	17 (41.5%)			
BMI grades	Average	24 (20%)	0	0	0.0001	0.0074	0.267
	Overweight	41 (34.2%)	18 (28.6%)	16 (39%)			
	Obese	55 (45.8%)	45 (71.4%)	25 (61%)			
Mean BMI (kg/m^2^)		21.8 ± 3.2	23.8 ± 1.9	24 ± 1.8	0.0001	0.0001	0.902
Tonsillar hypertrophy	Grade 1	2 (1.7%)	0	0	0.444	0.739	0.448
	Grade 2	31 (25.8%)	21 (33.3%)	9 (22%)			
	Grade 3	56 (46.7%)	24 (38.1%)	19 (46.3%)			
	Grade 4	31 (25.8%)	18 (28.6%)	13 (31.7%)			
Adenoid hypertrophy	No	10 (8.3%)	2 (3.2%)	0	0.272	0.212	0.079
	Grade 1	7 (5.8%)	3 (4.8%)	3 (7.3%)			
	Grade 2	46 (44.2%)	34 (53.9%)	12 (29.2%)			
	Grade 3	42 (35%)	19 (30.2%)	20 (48.9%)			
	Grade 4	15 (12.5%)	5 (7.9%)	6 (14.6%)			
Modified Mallampati score	Class 1	63 (52.5%)	34 (54%)	16 (39.1%)	0.074	0.0176	0.447
	Class 2	44 (36.7%)	15 (23.8%)	12 (29.2%)			
	Class 3	9 (7.5%)	12 (19%)	10 (24.4%)			
	Class 4	4 (3.3%)	2 (3.2%)	3 (7.3%)			

Data are presented as numbers, percentages, median, interquartile range [IQR], mean, standard deviation (±SD); AHI: apnea/hypoxia index; BMI: body mass index; *p*1 value indicates the significance of difference between patients had mild and moderate OSAS; *p*2 value indicates the significance of difference between patients had mild and severe OSAS; *p*3 value indicates the significance of difference between patients had moderate and severe OSAS; *p* < 0.05 indicates a significant difference; *p* > 0.05 indicates a non-significant difference.

**Table 2:** Outcome of the applied management of studied patients categorized according to OSAS severity.

Group	Variables (mean ± SD)	Mild OSAS (*n* = 120; 53.6%)	Moderate OSAS (*n* = 63; 28.1%)	Severe OSAS (*n* = 41; 18.3%)	Significance of Difference between Groups		
					*p*1	*p*2	*p*3
Frequency of residual OSAS		9 (7.5%)	11 (17.5%)	13 (31.7%)	0.04	0.001	0.092
Pediatric sleep questionnaire	Baseline	9.4 ± 1.9	9.7 ± 2.8	11.1 ± 2.7	0.692	0.002	0.005
	End of follow-up	1.1 ± 1	1.4 ± 1.3	1.9 ± 1.2	0.192	0.002	0.047
	*p*4 Value	<0.001	<0.001	<0.001			
Cognitive function Assessments	Baseline	9 ± 2.1	7.6 ± 1.8	6.9 ± 1.7	0.004	<0.001	0.096
	End of follow-up	10.1 ± 2.2	9.4 ± 1.9	9.1 ± 1.8	0.137	0.030	0.798
	*p*4 Value	0.001	<0.001	<0.001			
	% Of change	12.9 ± 10	25.2 ± 17.8	36.1 ± 19.9	0.002	<0.001	0.002
Cognitive outcome	Deteriorated	0	0	0	0.127	0.237	0.602
	No change	28 (23.3%)	10 (15.9%)	5 (12.2%)			
	Improved	92 (76.7%)	53 (84.1%)	36 (87.8%)			

Data are presented as numbers, percentages, median, interquartile range [IQR], mean, standard deviation (±SD); *p*1 value indicates the significance of difference between patients had mild and moderate OSAS; *p*2 value indicates the significance of difference between patients had mild and severe OSAS; *p*3 value indicates the significance of difference between patients had moderate and severe OSAS; *p*3 value indicates the significance of difference between baseline levels and levels estimated at the end of the follow-up; *p* < 0.05 indicates significant difference; *p* > 0.05 indicates non-significant difference.

**Table 3:** Serum levels of studied cytokines that were estimated at time of enrolment and at the end of the follow-up of patients categorized according to OSAS severity.

Serum Variables (mean ± SD)	Group	Mild OSAS (*n* = 120; 53.6%)	Moderate OSAS (*n* = 63; 28.1%)	Severe OSAS (*n* = 41; 18.3%)	Significance of Difference between Groups		
					*p*1	*p*2	*p*3
TNF-α	Baseline	6.14 ± 2.36	7.23 ± 1.6	8.38 ± 3.73	0.040	<0.001	0.031
	End of follow-up	3.15 ± 1.6	3.81 ± 1.75	3.91 ± 2.5	0.109	0.055	0.869
	*p*4 Value	<0.001	<0.001	<0.001			
	% Of change	47.2 ± 17.9	46.8 ± 20.7	54.4 ± 12.8	0.052	<0.001	0.0002
IL-6	Baseline	25.2 ± 10.5	30.8 ± 8.6	36 ± 9.4	0.005	<0.002	0.010
	End of follow-up	17.7 ± 7.9	19.7 ± 6.9	21.9 ± 7.8	0.324	0.008	0.252
	*p*4 Value	<0.001	<0.001	<0.001			
	% Of change	29.5 ± 10.6	36.2 ± 12.3	39.2 ± 13.5	0.002	0.005	0.328
IL-1β	Baseline	28.9 ± 8.2	23.2 ± 7.7	20.7 ± 9	0.232	<0.001	0.007
	End of follow-up	19 ± 7.1	13.7 ± 6.5	11 ± 6.3	0.057	<0.001	<0.001
	*p*4 Value	<0.001	<0.001	<0.001			
	% Of change	34.8 ± 13.8	40 ± 20.4	47.3 ± 20.2	0.146	0.002	0.207

Data are presented as numbers, percentages, mean, standard deviation ( ± SD); *p*1 value indicates the significance of difference between patients had mild and moderate OSAS; *p*2 value indicates the significance of difference between patients had mild and severe OSAS; *p*3 value indicates the significance of difference between patients had moderate and severe OSAS; *p*4 values indicates the significance of difference between baseline levels and levels estimated at the end of the follow-up; *p* < 0.05 indicates significant difference; *p* > 0.05 indicates non-significant difference.

**Table 4:** Spearman’s correlation between baseline values of the diagnostic variables of OSAS severity and baseline BMI and serum levels of the studied cytokines.

Variables	BMI		Serum TNF-α		Serum IL-6		Serum IL-1β	
	Rho	*p*	Rho	*p*	Rho	*p*	Rho	*p*
BMI	-	-	0.36	<0.001	0.117	0.08	0.181	0.007
AHI index	0.501	<0.001	0.314	<0.001	0.344	<0.001	0.297	<0.001
PSQ score	0.274	<0.001	0.167	0.012	0.183	0.006	0.091	0.175
NESPY score	0.218	−0.001	0.12	−0.074	0.219	−0.001	−0.114	0.09

BMI: body mass index; AHI: apnea/hypopnea index; PSQ: pediatric sleep questionnaire; TNF-α: tumor necrosis Factor-α; IL: interleukin; Rho: Spearman’s coefficient; *p* indicates the significance of Rho; *p* < 0.05 indicates significant difference; *p* > 0.05 indicates non-significant difference.

## Data Availability

The data presented in this study are available on request from the corresponding author. The data are not publicly available due to terms of privacy.
